# Multilocus Sequence Typing and Population Genetic Analysis of *Enterocytozoon bieneusi*: Host Specificity and Its Impacts on Public Health

**DOI:** 10.3389/fgene.2019.00307

**Published:** 2019-04-02

**Authors:** Wei Li, Lihua Xiao

**Affiliations:** ^1^Heilongjiang Key Laboratory for Zoonosis, College of Veterinary Medicine, Northeast Agricultural University, Harbin, China; ^2^College of Veterinary Medicine, South China Agricultural University, Guangzhou, China

**Keywords:** microsporidia, *Enterocytozoon bieneusi*, multilocus sequence typing, population genetics, host specificity, public health implication

## Abstract

Microsporidia comprise a large class of unicellular eukaryotic pathogens that are medically and agriculturally important, but poorly understood. There have been nearly 1,500 microsporidian species described thus far, which are variable in biology, genetics, genomics, and host specificity. Among those, *Enterocytozoon bieneusi* is the well-known species responsible for the most recorded cases of human microsporidian affections. The pathogen can colonize a broad range of mammals and birds and most of the animals surveyed share some genotypes with humans, posing a threat to public health. Based on DNA sequence analysis of the ribosomal internal transcribed spacer (ITS) and phylogenetic analysis, several hundreds of *E. bieneusi* genotypes have been defined and clustered into different genetic groups with varied levels of host specificity. However, single locus-based typing using ITS might have insufficient resolution to discriminate among *E. bieneusi* isolates with complex genetic or hereditary characteristics and to assess the elusive reproduction or transmission modes of the organism, highlighting the need for exploration and application of multilocus sequence typing (MLST) and population genetic tools. The present review begins with a primer on microsporidia and major microsporidian species, briefly introduces the recent advances on *E. bieneusi* ITS genotyping and phylogeny, summarizes recent MLST and population genetic data, analyzes the inter- and intragroup host specificity at the MLST level, and interprets the public health implications of host specificity in zoonotic or cross-species transmission of this ubiquitous fungus.

## Introduction

Microsporidia are an exceptional group of obligate intracellular protist-like fungi with hosts ranging from protists to mammals (Stentiford et al., 2016). A recent study indicated the organisms should be classified in a new, basal phylum, the Rozellomycota (or Cryptomycota) (Quandt et al., 2017). There existed ∼1,500 formally described microsporidian species in over 200 genera (Vavra and Lukes, 2013; Weiss and Becnel, 2014), among which *Enterocytozoon bieneusi* is an important etiological agent of diarrhea in AIDS patients (Matos et al., 2012). Although the colonization by *E. bieneusi* is predominantly related to immune suppression of hosts, a number of recent studies have reported its symptomatic and asymptomatic infections in immunocompetent individuals (Matos et al., 2012). Its emergence in multiple animal hosts signifies the necessity for genotype differentiation to reveal the origin and history of the pathogen (Thellier and Breton, 2008). Currently, the definition of *E. bieneusi* genotypes and assessment of their host specificity and zoonotic potential are greatly dependent on the analysis of single base-pair changes at the ribosomal internal transcribed spacer (ITS) (Santin and Fayer, 2011). The typing tool has been widely used in the surveys conducted in diverse areas around the world, which enables identification of several hundreds of genotypes that show varying degrees of host specificity (Guo et al., 2014; Karim et al., 2014b; Li et al., 2015). However, ITS genotyping might be insufficient for discriminating among genotypes with complex inheritance patterns (Feng et al., 2011). ITS genotyping seems to have also failed to substantiate any effects of genetic diversity on the geographic segregation of *E. bieneusi* (Widmer and Akiyoshi, 2010). Therefore, additional independent markers should be characterized to unravel subtle genetic variability among *E. bieneusi* isolates sampled in different hosts and environments (Feng et al., 2011). The use of multilocus sequence typing (MLST) tool is vital for elucidation of the epidemiology, population genetic structure and substructure, host specificity, mode of reproduction, and transmission pattern of this important fungus (Li et al., 2012, 2013, Li et al., 2016b; Karim et al., 2014c; Wan et al., 2016). The present review begins with a primer on microsporidia and major species, introduces the recent advances in the field of *E. bieneusi* typing and population genetics, and assesses the host specificity of *E. bieneusi* and the derived public health implications.

## A Primer on Microsporidia and Major Microsporidian Species

### Importance, Taxonomy, Host Specificity, and Zoonotic Potential of Microsporidia

Microsporidia have been known historically as parasitic pathogens of beneficial insects such as silkworms (*Nosema bombycis*) and honey bees (*Nosema apis* and *Nosema ceranae*) and fish (*Glugea anomala*). Chronic infections caused by the pathogens were a significant economic problem for silk farms, apiaries, and fisheries in the 1800s (Keeling and Mcfadden, 1998; Stentiford et al., 2013). Since the 1900s, microsporidia have been documented to be present in numerous other host taxa including humans (Mathis et al., 2005; Santin and Fayer, 2011; Stentiford et al., 2013). Clinical signs and symptoms of microsporidian infections vary widely depending on species of both host and pathogen, model of host-pathogen interaction, and host immune status (Didier et al., 2004; Mathis et al., 2005; Anane and Attouchi, 2010). Some members of microsporidia (e.g., *Encephalitozoon* spp. and *Nosema* spp.) are relevant to livestock veterinary medicine or pest control (Lomer et al., 2001; Maestrini et al., 2017). With the emergence of a large immunosuppressed population in the late 1980s, as a consequence of the AIDS/HIV pandemic, microsporidia (especially *E. bieneusi* and *Encephalitozoon* spp.) became prominent in the field of human medicine as important causes of diarrhea and systemic disease in HIV-infected patients. In addition, colonization of microsporidia has been repeatedly reported to negatively affect immunocompetent individuals in recent years (Didier and Weiss, 2006, 2011; Anane and Attouchi, 2010; Matos et al., 2012). The potential sources of human microsporidian infections have been evaluated, including other infected humans and animals and contaminated foods and water (Mathis et al., 2005; Anane and Attouchi, 2010; Izquierdo et al., 2011; Stentiford et al., 2016), although the concept of One Health has not been explicitly used in the survey and control of human microsporidiosis (Mwangi et al., 2016).

The classification of microsporidia has undergone many changes over the years, the origin of the organisms was historically fiercely debated (Keeling, 2009). Traditional systematics of microsporidia relied mainly on evolving knowledge of their morphologic characteristics, life-cycle patterns, and host-pathogen relationships, resulting in frequent modifications of the class, order, family, and species within the phylum (Larsson, 1986; Sprague et al., 1992; Franzen and Muller, 1999). The advent and employment of DNA sequencing technologies and phylogenetic approaches have revealed significant genetic divergence within and among microsporidan species and resolved some of the controversies concerning the classification of microsporidia, notably some species complexes that could not be differentiated by physical, biologic properties, or *in vitro* culture characteristics (Vossbrinck and Debrunner-Vossbrinck, 2005; Smith, 2009). Although almost 1,000 microsporidian species have been named in the same way as protists, an increasing amount of recent cladistic and genome evidences upheld a fungal origin of microsporidia (Keeling et al., 2000; Vivares et al., 2002; Lee et al., 2008; Keeling, 2009; Capella-Gutierrez et al., 2012; Quandt et al., 2017).

Microsporidian species can generally be grouped into generalists and specialists according to the ranges of phenotypic trait variation (Vavra and Lukes, 2013). Generalists commonly have broad host ranges and wide cell and tissue specificities, and they often cause opportunistic infections in higher vertebrates, among which *E. bieneusi* and three *Encephalitozoon* species (*E. cuniculi, E. hellem*, and *E. intestinalis*) are the best-known representatives with simple developmental cycles. They affect a wide variety of mammal hosts and together represent the most frequently reported causative agents of zoonotic microsporidian infections (Franzen, 2005; Mathis et al., 2005). In contrast, specialists are closely adapted for infections and developments in a single host species or a very narrow range of closely related host species, most of which would have no or minimal effects on public health (Mathis et al., 2005). The life cycles of specialists are typically somewhere in the range from relatively simple (e.g., *N. apis* and *N. ceranae* that complete their life cycles in one host individual) to extremely complex (e.g., *Amblyospora californica* that requires two successive host generations) (Andreadis, 2007; Gisder et al., 2011). Nevertheless, despite these advances, the issues on the host-range specificity of microsporidia and the associated phenotypic variations and public health implications need to be further elucidated by comparative genomics analysis.

### Genomics, Biology, and Genetics of Common Microsporidian Species

Intracellular parasitism commonly leads to gene loss and genome reduction, which enable adaptation of microbial pathogens to the selective pressures of different niches (Slamovits et al., 2004; Heinz et al., 2012; Jackson et al., 2016). This process might have shaped the exceedingly highly inter- and intraspecific diversity of microsporidia, facilitating an affection of the organisms for almost all animal taxa and eukaryotic cell types and concomitantly allowing various degrees of host range and specificity (Keeling and Fast, 2002; Keeling and Slamovits, 2004; Mathis et al., 2005).

Microsporidia have relatively compact genomes compared with other eukaryotes (Keeling and Slamovits, 2005). *E. intestinalis* has the smallest known eukaryotic nuclear genome of ∼2.3 megabases (Mb) (Corradi et al., 2010). Its sister, *E. cuniculi*, has also been noted for a high degree of genome reduction and compaction. The genome (∼2.9 Mb) of *E. cuniculi* contains nearly 2,000 densely packed genes located on 11 chromosomes (Katinka et al., 2001). Another relative species *E. hellem* has an estimated 2.5-Mb genome with few introns, reduced intergenic regions, and almost identical gene complements and chromosome arrangements to *E. cuniculi* and *E. intestinalis* (Pombert et al., 2012). Despite high concordance, a large amount of genetic variability has been revealed between the three *Encephalitozoon* species and among the ITS-based *E. cuniculi* genotypes I (rabbit strain), II (mouse strain), and III (dog strain) (Pombert et al., 2013; Pelin et al., 2016), which might be the consequences of evolutionary divergence and genetic selection for host-specific adaptation (Keeling and Fast, 2002; Corradi and Slamovits, 2011; Keeling and Corradi, 2011). These fascinating aspects make *E. cuniculi* a feasible model for comparative genomics.

Another generalist, *E. bieneusi*, possesses a relatively more expanded genome (∼6 Mb) than *E. cuniculi* (Akiyoshi et al., 2009; Keeling et al., 2010). Among 3,804 encoding genes predicted, 653 are homologous to those of *E. cuniculi*. The severe reduction of genes associated with energy generation and carbon metabolism in the *E. bieneusi* genome is indicative of the absence of a fully functional pathway to generate ATP from glucose, accounting for the strong host dependence of this pathogen (Akiyoshi et al., 2009; Keeling et al., 2010). The potential strategy for host exploitation of microsporidia might be associated with transporter proteins acquired from unrelated organisms by horizontal transfer (Corradi and Slamovits, 2011; Pombert et al., 2012; Heinz et al., 2014). The invasion and survival of microsporidia (e.g., *E. bieneusi* and *E. cuniculi*) in eukaryotic cells probably correlate with the ability of nucleoside transporters to catalyze the import of ATP from hosts and the enhancement of such processes by the specific interactions between the meront plasma membrane and host cell mitochondria (Tsaousis et al., 2008; Keeling et al., 2010). However, compacted genomes and massive adaptive gene losses might not be examples of the total microsporidian population. In fact, the genomic architecture of microsporidia varies remarkably among not only different species but also different genotypes within a given species (Keeling and Fast, 2002; Keeling and Slamovits, 2004; Slamovits et al., 2004). However, it is still largely unknown how the organisms have evolved such unique genomic, biological, and genetic features. Further efforts are required to clarify the association between the genomic architecture and host specificity of microsporidia and to elucidate the host-adaptation mechanisms of some important generalist species, notably *E. bieneusi*.

## *E. Bieneusi* Its Genotypes and Phylogenetic Groups

There is considerable genetic variation among *E. bieneusi* isolates sampled from humans, non-human primates (NHPs), porcines, ruminants, companion animals, equines, carnivores, rodents, lagomorphs, birds, water, etc., and great potential for zoonotic or cross-species transmission (Thellier and Breton, 2008; Henriques-Gil et al., 2010; Widmer and Akiyoshi, 2010; Santin and Fayer, 2011; Yang et al., 2015). Several hundreds of *E. bieneusi* genotypes have been defined based on the hypermutation of the ITS, which formed at least 10 phylogenetic groups that exhibit phenotypic heterogeneity (Thellier and Breton, 2008; Santin and Fayer, 2011, 2015; Guo et al., 2014; Karim et al., 2014c; Li et al., 2015; Zhong et al., 2017b). Widespread co-occurrence and high prevalence of some Group 1 genotypes (D, EbpC, type IV, etc.) in humans and a wide range of animals suggest potential zoonotic or interspecies transmission risks of those generalists (Thellier and Breton, 2008; Santin and Fayer, 2011). Yet, there seems to be a certain level of host specificity in Group 1 as inferred by the host range restriction of some of its members, such as genotypes A, EbpB, and PigEBITS3 (Thellier and Breton, 2008). Host specificity appears to be more common among Group 2 members, with different genotypes (e.g., BEB4, BEB6, I, and J) preferentially infecting bovines and ovines. In spite of this, Group 2 genotypes BEB4, BEB6, I, and J seem to have experienced host range expansion in recent years and could even cross host barriers and affect humans (Jiang et al., 2015; Fiuza et al., 2016). Most of the genotypes in the ITS groups other than Group 1 and Group 2 that display strong host specificity probably have limited or unknown public health importance, for instance, genotype PtEb IX is the frequent major contributor to *E. bieneusi* infections in dogs, but quite rarely seen in other hosts (Thellier and Breton, 2008; Santin and Fayer, 2011; Karim et al., 2014a; Jiang et al., 2015; Li et al., 2015; Ma et al., 2015). The findings need to be substantiated in MLST and population genetic studies on *E. bieneusi* strains isolated in multiple hosts and regions.

## Mlst and Host Adaptation of *E. Bieneusi*

### MLST

**FIGURE 1:**
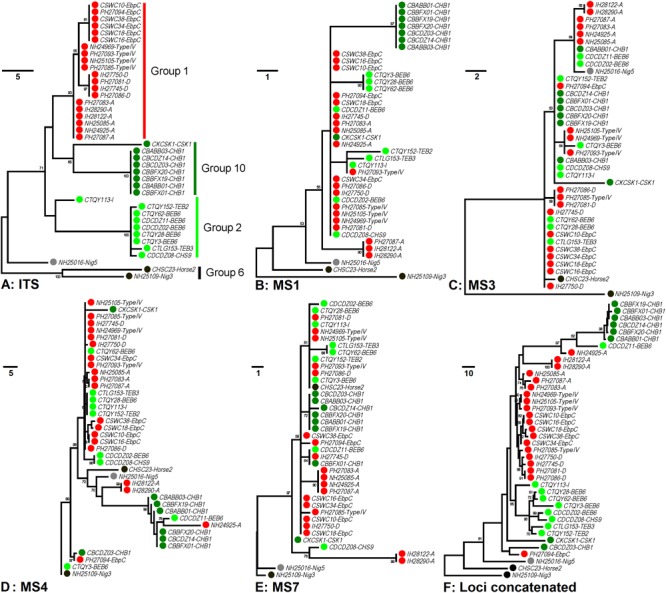
Phylogenetic analysis of individual and concatenated gene datasets of *Enterocytozoon bieneusi*. Phylogenies at loci ITS (panel **A**), MS1 (panel **B**), MS3 (panel **C**), MS4 (panel **D**), MS7 (panel **E**), and concatenated (panel **F**) were inferred by the maximum-parsimony method implemented in Mega 7 (http://www.megasoftware.net/). The reliability of cluster formation was evaluated by the bootstrap analysis with 1,000 replicates and the values generated greater than 50% were shown beside nodes. The colors (red, dark green, light green, black, and gray) of the circles specified before specimen codes represent the isolates are from ITS Groups 1 (*n* = 20), 10 (*n* = 8), 2 (*n* = 9), and 6 (*n* = 2) and an outlier group (*n* = 1), respectively. The first initials of isolate names, C, I, N, and P, indicate the isolates were sampled in China, India, Nigeria, and Peru, respectively. The letters B, D, H, K, S, and T followed demonstrate the isolates were sampled from bear, deer, human/horse (only human from India, Nigeria, and Peru was sampled, and horse from China likewise), kangaroo, swine, and takin, respectively. ITS genotypes are specified after specimen codes.

In order to increase discrimination to the level needed for subtyping *E. bieneusi*, a MLST tool was developed in 2011. Seven mini- and microsatellites were tested, four (MS1, MS3, MS4, and MS7) of which were determined to be appropriate for high-resolution typing of *E. bieneusi* (Feng et al., 2011). Patterns of genetic polymorphisms at the four loci are shown in Supplementary Table S1, with single-nucleotide polymorphisms (SNPs) and TGC, TAA, and TAC repeats found at MS1 (∼676 bp in length), SNPs and TA repeats at MS3 (∼537 bp), SNPs, insertions and deletions (INDELs), and GGTA repeats at MS4 (∼885 bp), and SNPs and TAA repeats at MS7 (∼471 bp). Phylogeny inferred by the neighbor-joining (NJ) method that considers only SNPs noted some similarities in cluster formation among the ITS and four mini- and microsatellites (Feng et al., 2011). Herein, genetic relationships among 40 known *E. bieneusi* isolates (Supplementary Table S2) were evaluated by using the maximum-parsimony (MP) method that considers both SNPs and INDELs (Figure 1). The ITS genotypes that the selected isolates belong to include several well-known generalists (e.g., D, EbpC, and type IV) as well as some others (e.g., BEB6, horse2, and I) that have restricted or extremely narrow host and geographic ranges (Santin and Fayer, 2011). All the isolates of ITS genotypes A, D, Nig3, Nig5, and type IV and one isolate of genotype EbpC were sampled from humans living in India, Nigeria, and Peru during 2000 to 2010 (Bern et al., 2005; Akinbo et al., 2012; Li et al., 2013), while sampling of the other isolates were conducted on multiple animal hosts (bear, deer, horse, kangaroo, swine, and takin) during 2012 to 2017 in China (Supplementary Table S2). The isolates analyzed formed four major phylogenetic groups (Groups 1, 2, 6, and 10) and an outlier group at the ITS locus (Figure 1A), in agreement with the findings based on the NJ analysis (Guo et al., 2014; Karim et al., 2014b; Li et al., 2015). However, the grouping patterns at loci MS1 (Figure 1B), MS3 (Figure 1C), MS4 (Figure 1D), and MS7 (Figure 1E) look quite intricate and completely different from each other and from that at the ITS (Figure 1A). Despite the discordance, phylogeny (Figure 1F) on the basis of combined multilocus contigs (∼2,300 bp) of all five markers did provide increased resolving power than when a unique marker was used (Figure 1A–E), as reflected by variation in the relative branch length.

The MLST tool was applied to investigate 72 *E. bieneusi* isolates sampled from adult AIDS patients residing in Peru for their genetic variations at all five loci, demonstrating that MS1 has the highest typing resolution and identifying 39 multilocus genotypes (MLGs) with varying genotype frequencies (1.4 to 36.1%) (Li et al., 2012). Likewise, additional 33 *E. bieneusi* isolates sampled from HIV-infected individuals in India (children) and Nigeria (adults) and five isolates from olive baboons in Kenya were analyzed, leading to recognition of a total of 27 MLGs that differed by regions and hosts. Most of the MLGs are also different from those identified in Peru (Li et al., 2013). Another study genotyped 197 *E. bieneusi* isolates originating from diarrheic children in Uganda at loci ITS, MS1, MS3, and MS7 and revealed the frequent occurrence of mixed infections in the study population (Widmer et al., 2013). Mixed infections have also been observed in diverse human *E. bieneusi* populations (Li et al., 2012, 2013). Combined analysis of genetic polymorphisms at all five loci identified 59 MLGs from 85 NHP-derived *E. bieneusi* isolates sampled in multiple regions of China (Karim et al., 2014c). The 44 MLGs generated from 101 pig-derived *E. bieneusi* isolates and 10 MLGs from 39 fur animal-derived isolates in China are distinct from those identified in primates (Li et al., 2016b; Wan et al., 2016). In addition, different levels of genetic diversity was observed within *E. bieneusi* isolated from domestic, wild, and zoo animals in China and within and among the genetic loci used, although most of the studies presented the typing data merely at some of the loci with the generated MLGs quite limited in number (Zhao et al., 2015; Deng et al., 2016a,b, 2017; Li et al., 2016a, 2017; Wang et al., 2016; Zhang et al., 2016; Zhong et al., 2017a,b). Despite substantial progress in MLST of *E. bieneusi*, there are still so many issues which need addressing, such as the genetic relationships among the MLGs reported thus far and the potential occurrence of host adaptation (refers to the capability of a pathogen to circulate and cause disease in a particular host population. It is commonly an indicator of the pathogen’s fitness or specificity of adaptation to its host environment, while might result in reduced occurrence of interspecies transmission of the pathogen) in this important fungus.

### Host Adaptation and Public Health Concern of Group 1 Genotypes

The first population genetic study analyzed both multilocus sequence- and allelic profile-based data derived from 72 human *E. bieneusi* isolates sampled in Peru and tested the intragenic and intergenic linkage disequilibrium (LD, refers to the status of a non-random association of alleles at various genetic loci. Between two loci with LD, the allele at one polymorphic locus can predict the allele of an adjacent polymorphic locus), the standardized index of association (*I*^S^_A_, that measures the LD between alleles in pair-wise combinations of markers with a zero or negative value indicative of a randomly mating population and a predicted value greater than zero indicative of a non-panmictic population structure), Fu’s neutrality, and genetic recombination event (Rm) to assess the population structure. The strong intragenic LD (|*D*′|*Y* = 1.0007 – 0.0432*X*; where *Y* is the LD value, and *X* is the nucleotide distance in kb), significant pair-wise intergenic LD among all pairs of markers (*p* < 0.001 for all comparisons), *I*^S^_A_ = 0.7043, lack of neutrality, and limited genetic recombination detected are evidences for a clonal structure in the population (refers to a population of organisms with a common origin. It is characterized by strong LD among genetic loci and frequently very little or no genetic diversity among isolates prevalent in the host at any given time) (Li et al., 2012). In another study, a number of *E. bieneusi* isolates sampled from humans in India and Nigeria and captive baboons in Kenya, together with some randomly selected Peruvian isolates were analyzed for population genetic characteristics. Clonality appears widespread among the three human *E. bieneusi* populations in India (intragenic LD ≈ 1.0021, significant intergenic LD, and *I*^S^_A_ = 0.4835), Nigeria (intragenic LD ≈ 0.9647, significant intergenic LD, and *I*^S^_A_ = 0.5746), and Peru (intragenic LD ≈ 0.9993, significant intergenic LD, and *I*^S^_A_ = 0.3073), and absence of significant recombination was determined (Li et al., 2013). Despite the possibility of recombination in those populations, it is too weak to break the prevalent pattern of clonal population structure as previously suggested (Tibayrenc and Ayala, 2012). The same typing tool and data processing and analysis strategies were used to survey *E. bieneusi* isolates derived from NHPs in China, which showed similar findings to those found in humans (Karim et al., 2014c). However, the results must be substantiated using isolates from the hosts other than primates. Strikingly, two *E. bieneusi* populations from pigs and fur-bearing animals in China were shown undergoing clonal evolution with strong and significant LD and rare Rms detected, consistent with the observations in primates (Li et al., 2016b; Wan et al., 2016).

**FIGURE 2:**
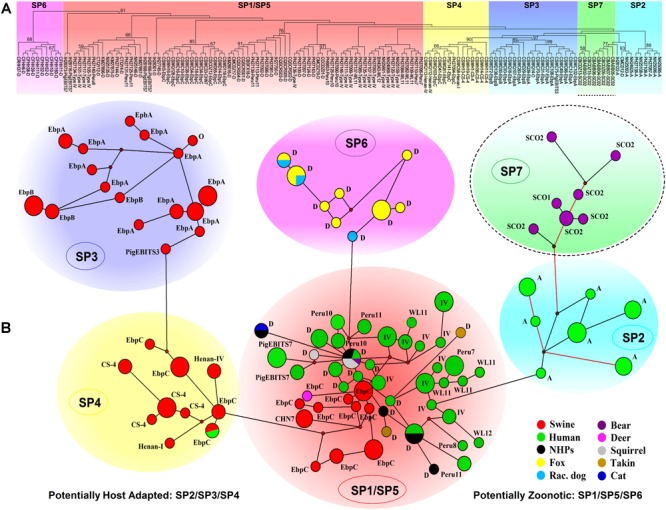
Multilocus phylogeny and haplotype network of *Enterocytozoon bieneusi* ITS Group 1 isolates sampled in various hosts and locations. All the *E. bieneusi* isolates (*n* = 246, Supplementary Table S2) that were previously genotyped as Group 1 members were included in the analyses except for five isolates (2 for genotype IH and 3 for genotypes D, horse1, and Nig2) with multilocus sequences significantly divergent from the other Group 1 members. The naming of subpopulations is based chiefly on that described previously (Wan et al., 2016). SP7 is highlighted with a dashed border as its host specificity and zoonotic potential are poorly understood. Panel **A**: Maximum-parsimony phylogeny of 138 unique multilocus genotypes (MLGs) generated considering both single-nucleotide polymorphisms (SNPs) and insertions and deletions from the total isolates using Mega 7 (http://www.megasoftware.net/). The first initials of isolate names, B, C, I, K, N, and P, indicate the isolates were sampled in Brazil, China, India, Kenya, Nigeria, and Peru, respectively. The letters B, C, D, F, H, M, Q, R, S, and T followed indicate the isolates were sampled from bear, cat, deer, fox, human, monkey, squirrel, raccoon dog, swine, and takin, respectively. ITS genotypes are labeled at the ends. Bootstrap values (1,000 replicates) above 50% are shown at the nodes. Panel **B**: Genetic network analysis of all the 246 isolates using the median-joining method implemented in Network 4.6.1.1 (http://www.fluxus-engineering.com/sharenet_rn.htm). The colors in circles correspond to different hosts sampled. The size of the circles is proportional to the frequency of each of the 98 MLGs obtained with consideration of only SNPs. ITS genotypes are labeled beside the circles. Genotype type IV is abbreviated as IV for better presentation and non-human primates as NHPs likewise. The branches connecting MLGs have a length proportional to the number of SNPs, while the red branches having pairwise differences above 12 SNPs are shortened for better presentation.

The ITS-based host range analysis has demonstrated the potential existence of host adaptation in Group 1 (Thellier and Breton, 2008). The MLST-based population genetic analysis in a global context would be more precise in assessing subgroup formation and host-specific difference and public health implication of subgroups. Herein, we executed extensive multilocus phylogenetic analysis and genetic network analysis using almost all *E. bieneusi* Group 1 isolates with nucleotide sequence data available at all five loci (Supplementary Table S2) and presented the genetic relationship of MLGs of various host origins in Figure 2, facilitating formation of seven subpopulations (SP1 to SP7). SP1 (reported as sub-population 2) and SP2 (reported as sub-population 1) originate from subdivision of the overall primate *E. bieneusi* population via phylogenetic and structural analyses. The varied levels of LD (SP1: incomplete intragenic LD ≈ 0.9473 and *I*^S^_A_ = 0.1191; SP2: complete intragenic LD ≈ 1.0165 and *I*^S^_A_ = 0.3729) and recombination potential (far fewer Rms in SP2 than SP1) between the two subpopulations coupled with Wright’s fixation index (a measure of population divergence, with zero indicating similar polymorphisms across all markers and one showing a high level of divergence between populations, *F*_ST_ = 0.391) analysis confirmed the segregation of SP1 and SP2 and revealed their different genetic structures and transmission routes. SP1 with a clonal structure contains mainly isolates identified at ITS as zoonotic genotypes (D, type IV, etc.), whereas SP2 with an epidemic structure (refers to a population characterized by potential recombination in which a particular successful clone may be generated, becoming the predominant one for an extended period) comprises isolates defined at ITS as anthroponotic genotype A. In spite of the sequence diversity of MLGs as mentioned earlier, no significant geographic difference in population genetic structure was ascertained (Li et al., 2012, 2013). While ITS Group 1 was commonly considered at potential risk for zoonotic or cross-species transmission, a level of host specificity was suggested in SP2 as inferred by the appearance of complete LD and quite limited recombination as well as the epidemic expansion of several advantageous MLGs in the subpopulation, signifying potential occurrence of host adaptation in *E. bieneusi* (Li et al., 2012, 2013; Karim et al., 2014c).

Nevertheless, it could be questioned whether the observations in primate *E. bieneusi* populations are also present in other host species. Another three subpopulations, SP3 to SP5 (Figure 2), were originally defined in the overall pig *E. bieneusi* population by sub-structural analyses, with genetic segregation of the isolates at some geographic level. They are divergent from the primate-derived SP1 and SP2 to some extent. The measurement of population divergence among SP1 to SP5 by analysis of *F*_ST_ and gene flow (*Nm*) showed a close genetic relationship between SP1 and SP5 (*F*_ST_ = 0.185 and *Nm* = 1.10) and the differentiation of SP1/SP5 from SP2/SP3/SP4 (pair-wise *F*_ST_ values of at least 0.342 and *Nm* values of at most 0.48). Genetic network analysis showed directed evolution of SP5 to SP3/SP4 and SP1 to SP2 (Wan et al., 2016). That SP2 to SP4 harbor isolates mostly belonging to ITS genotypes A, EbpA, and EbpB with restricted host and geographical ranges, and the strong LD (intragenic LD for SP2/SP3/SP4 ≈ 1.0165/0.9724/0.9844 and *I*^S^_A_ for SP2/SP3/SP4 = 0.3729/0.4709/0.4866) and a quite small number of Rms examined, are indicators of potential host adaptation in the three subpopulations (Wan et al., 2016). In contrast, SP1 and SP5 consist of isolates majorly pertaining to zoonotic ITS genotypes D, type IV, and EbpC that have a broad host range and a worldwide geographic distribution. The reduced LD (intragenic LD for SP1/SP5 ≈ 0.9473/0.9399 and *I*^S^_A_ for SP1/SP5 = 0.1191/0.2535) and enhanced genetic diversity in SP1 and SP5 might be consequences of host range expansion and ability to adapt to new host environment (Wan et al., 2016). Distinction of the fur animal *E. bieneusi* population SP6 (made up of zoonotic genotype D isolates) from SP1 to SP5 was perspicuously seen in Figure 2. It remains obscure if host adaptation occurred in SP6 because of a too low sample size, however, a high degree of genetic diversity and a clonal structure demonstrate the potential cross-species capacity of this subpopulation (Li et al., 2016b). It is noteworthy that several *E. bieneusi* isolates (belonging to Group 1 genotypes SC01 and SC02) obtained from captive bears in China (Li et al., 2016a; Deng et al., 2017) formed a new subpopulation SP7, which is genetically linked to human-adapted SP2 and highly separate from SP1 and SP5 with cross-species potential (Figure 2). Additionally, several sporadic isolates originating from cat (1 genotype D isolate), bear (1 genotype D isolate), deer (1 genotype EbpC isolate), takins (3 genotype D isolates), and squirrels (5 genotype D isolates) were clustered into the zoonotic SP1/SP5, upholding zoonotic nature of some *E. bieneusi* isolates (Supplementary Table S2 and Figure 2).

The above findings can be explained by three characteristics that commonly exist in a clonal population (Tibayrenc and Ayala, 2002): 1) genetic exchange could be occurring infrequently, 2) the population could be separated into genetically isolated subdivisions, and 3) there may be a clonal endemicity in the population. It has been potentially remarked that, high diversity might weaken host specificity of eukaryotic microbial pathogens and enable genetic selection to cope with diverse host species and responses to environmental challenges (Heitman, 2010). Therefore, the isolates in SP1, SP5, and SP6 that have high genetic diversity and undergo predominantly clonal propagation might be responsible for zoonotic or cross-species transmission and thus have public health importance. In contrast, the isolates in SP2, SP3, and SP4 with an epidemic structure or host-adapted features might mainly affect particular hosts and initiate allopatric speciation, as deduced from over-representation of specific MLGs in some of the subpopulations (Li et al., 2012, 2013, 2016b; Wan et al., 2016). In conclusion, the collective data have demonstrated the presence of clonality, population differentiation, and potential host adaptation of *E. bieneusi* in different hosts.

Population genetics and comparative genomics have provided some novel insights into evolutionary trajectory of the machinery for sexual reproduction and meiosis of some microbes (Lee et al., 2010b; Tibayrenc and Ayala, 2014). Alternation between sexual and asexual phases has been documented in many parasitic microorganisms (Tibayrenc and Ayala, 2002; Haag et al., 2013a). It is remarkable that asexual mode of reproduction would enable epidemic expansion of microbe populations, while sexuality holds its importance in promoting genetic variation, facilitating the spread of adaptive mutations, and increasing evolutionary fitness in response to environmental challenges (Heitman, 2006, 2010). However, still we know relatively little about the population genetic traits and reproduction and transmission modes for most species of microsporidia, including *E. bieneusi*. Early assessment of recondite reproductive modes in microsporidia is mostly dependent on the patterns of the cell structure and life cycle development (Chen and Barr, 1995; Canning et al., 1999; Andreadis, 2007). It has been indicated that single-host microsporidian species commonly possess obligate asexuality, while those experiencing host alterations in their life cycles often undergo sexual reproduction (Andreadis, 2007). Some microsporidian species (e.g., *N. apis* and *Nosema bombi*) are considered to be asexual since they have no monokaryotic stage throughout life cycle and lack concerted evolution of rRNA genes in a single spore and even in a single nucleus (Tay et al., 2005; O’mahony et al., 2007). However, molecular phylogenetic data on several *Nosema* and *Vairimorpha* species have provided no evidence for ancient asexuality, instead indicating that sex has been lost on multiple and independent occasions (Ironside, 2007). Genome sequencing and comparison of eight geographically segregated *N. ceranae* isolates offered evidence for clonality and polyploidy in at least one isolate (Pelin et al., 2015). Genomic expansion of *N. bombycis* compared to its congener *Nosema antheraeae* was inferred to be related to enhanced infection ability and expanded host range of the former (Pan et al., 2013). A shared conserved sex-determining locus and a high degree of genome-wide synteny between the three microsporidian species (*E. bieneusi, E. cuniculi*, and *Antonospora locustae*) and zygomycetes constitute evidence for the occurrence of sex in microsporidia (Lee et al., 2008, 2010a). Genome-wide DNA polymorphism analysis indicated the existence of sexuality in *Nematocida* that naturally infects *Caenorhabditis nematodes* and showed that genetic diversity could arise concomitantly under positive selection during host adaptation (Cuomo et al., 2012). Comparison of genomic and RNA-seq data between *Edhazardia aedis* and *Vavraia culicis* showed different evolutionary trajectories of microsporidia-mosquito interactions and adaptive processes (Desjardins et al., 2015). A recent population genetic study analyzed the mode of reproduction of *Daphnia*-harboring organism *Hamiltosporidium* and concluded the presence of sexuality, but the sexual process could be cryptic or lost (Haag et al., 2013b). Analysis of population genomic traits of a set of *Spraguea lophii* isolates sampled from fish revealed geographic segregation in genetic diversity and identified some contributors to evolutionary novelty and adaptation (Williams et al., 2016). It has been generally accepted that sexual reproduction might occur in microsporidia, while some species or genotypes could have switched from sexuality to obligate asexuality (Ironside, 2007; Haag et al., 2013b). This phenomenon is supported by the existence of both clonal and epidemic genetic structures in *E. bieneusi* populations of human and pig origins (Li et al., 2012, 2013; Wan et al., 2016). It has also been proposed that sex might be present in *E. bieneusi*, although sometimes lost or cryptic and that the organism could switch to obligate sexuality when an epidemic population structure arises (Wan et al., 2016). Despite those findings, it remains highly doubtful whether microsporidia undergo mating and a meiotic cycle and whether and how the current obligately asexual species have evolved from their sexual ancestors.

### Host Adaptation in Other ITS Phylogenetic Groups

**FIGURE 3:**
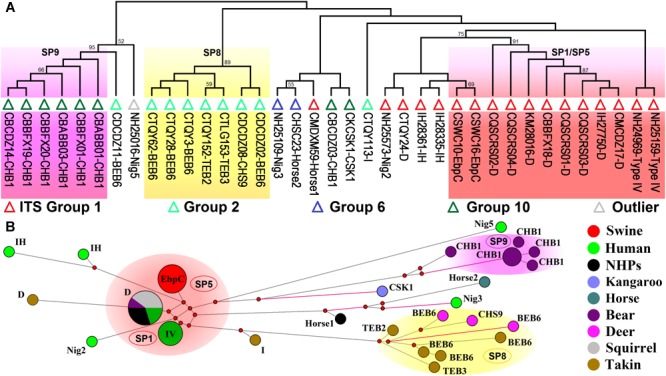
Multilocus phylogeny and haplotype network of *Enterocytozoon bieneusi* isolates from different hosts and geographic origins that belong to Group 1 and several other ITS groups. All the *E. bieneusi* isolates (*n* = 20) that belong to the ITS groups other than Group 1 were included in the analyses and some of the Group 1 isolates (*n* = 27) with genotypes being D, EbpC, type IV, horse1, IH, and Nig2 were selected for comparative analysis (Supplementary Table S2). Panel **A**: Maximum-parsimony phylogeny of 37 unique multilocus genotypes (MLGs) generated considering both single-nucleotide polymorphisms (SNPs) and insertions and deletions from the total isolates using Mega 7 (http://www.megasoftware.net/). The first initials of isolate names, C, I, and N, indicate the isolates were sampled in China, India, and Nigeria, respectively. The letters B, D, H, K, M, Q, S, and T followed indicate the isolates were sampled from bear, deer, human/horse (only human from India and Nigeria was sampled, and horse from China likewise), kangaroo, monkey, squirrel, swine, and takin, respectively. ITS genotypes are labeled at the ends. ITS Groups 1, 2, 6, and 10 and an outlier group that the isolates belong to were indicated by red, light green, blue, dark green, and gray triangles, respectively. Bootstrap values above 50% from 1,000 pseudoreplicates are displayed. Panel **B**: Median-joining network analysis of all the total 47 isolates using Network 4.6.1.1 (http://www.fluxus-engineering.com/sharenet_rn.htm). The colors in circles represent various host sources. The size of the circles is proportional to the frequency of each of the 26 MLGs obtained based on only SNPs. ITS genotypes are labeled beside the circles. Genotype type IV is abbreviated as IV for better presentation and non-human primates as NHPs likewise. The black branches connecting MLGs have a length proportional to the SNPs, while the red branches having pairwise differences greater than 12 SNPs are shortened for better presentation.

Analyses of host ranges of *E. bieneusi* ITS genotypes have implied relatively stronger host specificity in the ITS groups other than Group 1 (Thellier and Breton, 2008). MLST might provide more reliable data that help elucidate molecular basis of *E. bieneusi* host adaptation. We performed multilocus phylogenetic analysis and genetic network analysis using 19 *E. bieneusi* isolates that belong to ITS Group 2 (genotypes BEB6, CHS9, I, TEB2, and TEB3), Group 6 (genotypes horse2 and Nig3), and Group 10 (genotypes CHB1 and CSK1), an outlier genotype Nig5, and 27 Group 1 isolates with genotypes being D, EbpC, type IV, horse1, IH, and Nig2 (Supplementary Table S2 and Figure 3). The isolates formed three main clusters, one containing zoonotic MLGs from SP1/SP5, one named SP8 containing MLGs with ITS genotypes belonging to Group 2, and the rest named SP9 containing MLGs belonging to the ITS Group 10 genotype CHB1 (Figure 3). SP8 and SP9 may represent two subpopulations that are specific to ruminants (takins and deer) and bears, respectively, as indicated by their significant departure from SP1/SP5 (Figure 3) and the potential occurrence of directed evolution (from cross-species or zoonotic MLGs to host-adapted MLGs) in *E. bieneusi*, as proposed previously (Wan et al., 2016). Some MLGs belonging to ITS genotypes CSK1, horse2, Nig3, and Nig5 might also be adapted to specific hosts because they are highly divergent from those included in SP1/SP5, and their host ranges are quite limited (Figure 3; Wan et al., 2016; Zhong et al., 2017b). Yet, it is very difficult to assess the levels of host specificity of several relatively discrete MLGs with ITS genotypes being I, IH, horse1, Nig2, etc. (Figure 3). To date, very limited MLST data have been generated for *E. bieneusi* isolates belonging to Groups 2 to 10 since the existing tool has typically failed to amplify mini- and microsatellites in those isolates. This may be attributable to that the online locus searches of short (≤6 bp) or long (>6 bp) tandem repeat units using software Tandem Repeats Finder^1^, were run on the genomic scaffolds or contigs derived merely from a single *E. bieneusi* isolate (Feng et al., 2011). Thus, it is still highly desirable for typing markers suitable for all existing *E. bieneusi* isolates to uncover more complex factors that modulate the cross-species ability and host adaptation of the pathogen. Comparative genomic analysis of various *E. bieneusi* isolates that display phenotypic differences would provide useful information on the exploration of new polymorphic genetic markers.

## Conclusion

We briefly introduce taxonomy, biology, genetics, and genomics of microsporidia to understand the derived host-adapted characteristics and public health and veterinary importance. Microsporidia differ significantly from species to species in their life cycle, biology, genome organization, and host specificity, which makes the single-celled organisms ideal to elaborate the host-pathogen evolution and transmission. *E. bieneusi* has shrinking genome, greatly reduced biological complexity, and robust host dependence. Extreme intraspecific genetic variability and varied levels of host specificity were ascertained within *E. bieneusi* at the ITS and MLST levels. Genetic analysis of population structure and substructure demonstrates that *E. bieneusi* might be transmitted differently in a given host.

The data would be helpful in elucidating the host-adaptive mechanism of *E. bieneusi* and its implications to zoonotic and interspecies transmission of this ubiquitous pathogen. However, our understanding of the epidemiology and population genetic traits of the pathogen is still quite superficial. Additional *E. bieneusi* MLST and genomic data from other hosts and geographic areas are needed for in-depth assessment of the potential role of either host species or geography in genetic clustering of MLGs, host specificity, virulence or clinical presentations, and transmission risks.

## Author Contributions

WL and LX conceptualized, read, and approved the manuscript. WL collected, analyzed the data, elaborated the figures, and drafted the manuscript. LX revised the manuscript.

## Conflict of Interest Statement

The authors declare that the research was conducted in the absence of any commercial or financial relationships that could be construed as a potential conflict of interest.

## Footnotes

1 http://tandem.bu.edu/trf/trf.html
