# Supplementary material for: Multilocus Sequence Typing and Population Genetic Analysis of Enterocytozoon bieneusi: Host Specificity and Its Impacts on Public Health
Source: Front Genet. 2019 Apr 2;10:307. doi: 10.3389/fgene.2019.00307 (PMC6454070; doi:10.3389/fgene.2019.00307)
Supplement: Supplementary file 1 [file Table_1.doc]

| **Target ^(1)^ (PCR type)** | **Primer sequence (5’ to 3’)** | **Size (bp)** | **Polymorphism ^(2)^** | **Reference** |
| --- | --- | --- | --- | --- |
| MS1 (nested) | **F1**: CAAGTTGCAAGTTCAGTGTTTGAA  **R1**: GATGAATATGCATCCATTGATGTT  **F2**: TTGTAAATCGACCAAATGTGCTAT  **R2**: GGACATAAACCACTAATTAATGTAAC | ~676 | SNPs and trinucleotide TGC, TAA, and TAC repeats |  |
| MS3 (nested) | **F1**: CAAGCACTGTGGTTACTGTT  **R1**: AAGTTAGGGCATTTAATAAAATTA  **F2**: GTTCAAGTAATTGATACCAGTCT  **R2**: CTCATTGAATCTAAATGTGTATAA | ~537 | SNPs and dinucleotide TA repeats |  |
| MS4 (nested) | **F1**: GCATATCGTCTCATAGGAACA  **R1**: GTTCATGGTTATTAATTCCAGAA  **F2**: CGAAGTGTACTACATGTCTCT  **R2**: GGACTTTAATAAGTTACCTATAGT | ~885 | SNPs, INDELs, and tetranucleotide GGTA repeats |  |
| MS7 (nested) | **F1**: GTTGATCGTCCAGATGGAATT  **R1**: GACTATCAGTATTACTGATTATAT  **F2**: CAATAGTAAAGGAAGATGGTCA  **R2**: CGTCGCTTTGTTTCATAATCTT | ~471 | SNPs and trinucleotide TAA repeats |  |

**TABLE S1** Genetic markers and PCR primers used in multilocus sequence typing of *Enterocytozoon bieneusi.*

***^(1)^****MS1/3/7, microsatellite loci 1/3/7; MS4, minisatellite locus 4.* ***^(2)^****SNPs, single-nucleotide polymorphisms; INDELs, insertions and deletions.*

**REFERENCES**

Feng, Y., Li, N., Dearen, T., Lobo, M.L., Matos, O., Cama, V., and Xiao, L. (2011). Development of a multilocus sequence typing tool for high-resolution genotyping of *Enterocytozoon bieneusi*. *Appl Environ Microbiol* 77**,** 4822-4828.

Li, W., Cama, V., Feng, Y., Gilman, R.H., Bern, C., Zhang, X., and Xiao, L. (2012). Population genetic analysis of *Enterocytozoon bieneusi* in humans. *Int J Parasitol* 42**,** 287-293.

Wan, Q., Xiao, L., Zhang, X., Li, Y., Lu, Y., Song, M., and Li, W. (2016). Clonal evolution of *Enterocytozoon bieneusi* populations in swine and genetic differentiation in subpopulations between isolates from swine and humans. *PLoS Negl Trop Dis* 10**,** e0004966.
