# Supplementary material for: Multilocus Sequence Typing and Population Genetic Analysis of Enterocytozoon bieneusi: Host Specificity and Its Impacts on Public Health
Source: Front Genet. 2019 Apr 2;10:307. doi: 10.3389/fgene.2019.00307 (PMC6454070; doi:10.3389/fgene.2019.00307)
Supplement: Supplementary file 2 [file Table_2.doc]

**TABLE S2 *Enterocytozoon bieneusi* isolates used in multilocus sequence typing in this review.**

| **Host** | **Location** | **Code (no. of specimens)** | ***ITS phylogenetic groups*: genotype (no. of isolates)** | **Isolates (ITS genotype): GenBank accession no. (MS1, MS3, MS4, MS7) ^(1)^** | **Reference** |
| --- | --- | --- | --- | --- | --- |
| Human | Brazil | BH (1) | ***1***: WL12 (1) | BH4343 (WL12): JF951429, HQ615895, HQ615909, HQ615925 |  |
|  | India | IH (16) | ***1***: A (3), D (5), PigEBITS7 (6), IH (2) | Nucleotide sequences of MS1, MS3, MS4, and MS7 are available in the GenBank database under the accession numbers JQ991371 to JQ991386, JQ991409 to JQ991424, JQ991447 to JQ991462, and JQ991485 to JQ991500, respectively. |  |
|  | Nigeria | NH (17) | ***1***: A (6), type IV (6), D (2), Nig2 (1); ***6***: Nig3 (1); ***Outlier***: Nig5 (1) | Nucleotide sequences of MS1, MS3, MS4, and MS7 are available in the GenBank database under the accession numbers JQ991392 to JQ991408, JQ991430 to JQ991446, JQ991468 to JQ991484, and JQ991506 to JQ991522, respectively. |  |
|  | Peru | PH (72) | ***1***: A (30), type IV (16), D (8), Peru7 (5), Peru11 (4), WL11 (4), Peru10 (3), EbpC (1), Peru8 (1) | Nucleotide sequences of MS1, MS3, MS4, and MS7 are available in the GenBank database under the accession numbers JN637477 to JN637548, JN637549 to JN637620, JN637621 to JN637692, and JN637693 to JN637764, respectively. |  |
|  | Peru | PH (1) | ***1***: Peru10 (1) | PH6653 (Peru10): HQ615889, HQ615893, HQ615907, HQ615920 |  |
| Nonhuman primate | China | CM (1) | ***1***: D (1) | CMCD12 (D): KF305597, KF305611, KF305615, KF305628 |  |
|  | China | CM (2) | ***1***: D (2) | CMCDZ17 (D): KU871874, KU871910, KU871944, KU871977; CMCDZ19 (D): KU871876, KU871911, KU871945, KU871979 |  |
|  | China | CM (1) | ***1***: Horse1 (1) | CMDXM59 (Horse1): KX905213, KX905233, KX905234, KX905240 |  |
|  | Kenya | KM (5) | ***1***: D (5) | Nucleotide sequences of MS1, MS3, MS4, and MS7 are available in the GenBank database under the accession numbers JQ991387 to JQ991391, JQ991425 to JQ991429, JQ991463 to JQ991467, and JQ991501 to JQ991505, respectively. |  |
| Swine | China | CS (101) | ***1***: CHN7 (3), CS-4 (27), EbpA (22), EbpB (12), EbpC (32), Henan-I (1), Henan-IV (2), O (1), PigEBITS3 (1) | Nucleotide sequences of MS1, MS3, MS4, and MS7 are available in the GenBank database under the accession numbers KU212402 to KU212502, KU212503 to KU212603, KU212604 to KU212704, and KU212705 to KU212805, respectively. |  |
|  | Peru | PS (1) | ***1***: EbpC (1) | PS7141 (EbpC): HQ615888, HQ615900, HQ615905, HQ615919 |  |
| Deer | China | CD (4) | ***1***: EbpC (1); ***2***: BEB6 (2), CHS9 (1) | CDCDZ12 (EbpC): KU871869, KU871905, KU871939, KU871973; CDCDZ02 (BEB6): KU871861, KU871898, KU871932, KU871967; CDCDZ11 (BEB6): KU871868, KU871904, KU871938, KU871972; CDCDZ08 (CHS9): KU871866, KU871902, KU871936, KU871971 |  |
| Takin | China | CT (9) | ***1***: D (3); ***2***: BEB6 (3), I (1), TEB2 (1), TEB3 (1) | CTQY4 (D): KR048552, KR048572, KR048585, KR048608; CTQY12 (D): KR048542, KR048564, KR048582, KR048600; CTQY24 (D): KR048554, KR048565, KR048591, KR048616; CTQY3 (BEB6): KR048548, KR048571, KR048590, KR048610; CTQY28 (BEB6): KR048546, KR048560, KR048579, KR048597; CTQY62 (BEB6): KR048550, KR048562, KR048595, KR048606; CTQY113 (I): KR048558, KR048575, KR048586, KR048612; CTQY152 (TEB2): KR048545, KR048577, KR048587, KR048604; CTLG153 (TEB3): KR048557, KR048570, KR048588, KR048609 |  |
| Cat | China | CC (1) | ***1***: D (1) | CCCDZ01 (D): KU871860, KU871897, KU871931, KU871966 |  |
| Horse | China | CH (1) | ***6***: Horse2 (1) | CHSC23 (Horse2): KX276674, KX276679, KX276694, KX276704 |  |
| Bear | China | CB (10) | ***1***: SC01 (1), SC02 (3), D (1); ***10***: CHB1 (5) | CBBFX07 (SC01): KU871885, KU871919, KU871954, KU871988; CBCDZ05 (SC02): KU871864, KU871900, KU871934, KU871969; CBCDZ13 (SC02): KU871870, KU871906, KU871940, KU871974; CBCDZ15 (SC02): KU871872, KU871908, KU871942, KU871976; CBBFX18 (D): KU871894, KU871928, KU871963, KU871998; CBCDZ03 (CHB1): KU871862, KU871899, KU871933, KU871968; CBCDZ14 (CHB1): KU871871, KU871907, KU871941, KU871975; CBBFX01 (CHB1): KU871879, KU871913, KU871948, KU871982; CBBFX19 (CHB1): KU871895, KU871929, KU871964, KU871999; CBBFX20 (CHB1): KU871896, KU871930, KU871965, KU872000 |  |
|  | China | CB (6) | ***1***: SC02 (4); ***10***: CHB1 (2) | CBABB02 (SC02): KY021398, KY021404, KY021406, KY021413; CBABB04 (SC02): KY021399, KY021404, KY021408, KY021413; CBABB05 (SC02): KY021403, KY021405, KY021408, KY021412; CBABB06 (SC02): KY021399, KY021404, KY021408, KY021413; CBABB01 (CHB1): KY021400, KY021403, KY021407, KY021410; CBABB03 (CHB1): KY021397, KY021405, KY021407, KY021411 |  |
| Fox | China | CF (13) | ***1***: D (13) | Nucleotide sequences of MS1, MS3, MS4, and MS7 are available in the GenBank database under the accession numbers KU306407 to KU306517. |  |
| Raccoon dog | China | CR (3) | ***1***: D (3) | CRHH16 (D): KU306439, KU306450, KU306464, KU306497; CRHH37 (D): KU306437, KU306462, KU306469, KU306502; CRHH88 (D): KU306433, KU306451, KU306474, KU306495 |  |
| Squirrel | China | CQ (5) | ***1***: D (5) | CQSCRS01 (D): KX259505, KX259512, KX259515, KX259519; CQSCRS02 (D): KX259506, KX259512, KX259515, KX259518; CQSCRS03 (D): KX259507, KX259512, KX259515, KX259518; CQSCRS04 (D): KX259508, KX259514, KX259515, KX259518; CQSCRS05 (D): KX259509, KX259514, KX259517, KX259518 |  |
| Kangaroo | China | CK (1) | ***10***: CSK1 (1) | CKCSK1 (CSK1): KY706117, KY706118, KY706123, KY706125 |  |

***^(1)^****MS1/3/7, microsatellite loci 1/3/7; MS4, minisatellite locus 4.*

**REFERENCES**

Deng, L., Li, W., Yu, X., Gong, C., Liu, X., Zhong, Z., Xie, N., Lei, S., Yu, J., Fu, H., Chen, H., Xu, H., Hu, Y., and Peng, G. (2016a). First report of the human-pathogenic *Enterocytozoon bieneusi* from red-bellied tree squirrels (*Callosciurus erythraeus*) in Sichuan, China. *PLoS One* 11**,** e0163605.

Deng, L., Li, W., Zhong, Z., Gong, C., Cao, X., Song, Y., Wang, W., Huang, X., Liu, X., Hu, Y., Fu, H., He, M., Wang, Y., Zhang, Y., Wu, K., and Peng, G. (2017). Multi-locus genotypes of *Enterocytozoon bieneusi* in captive Asiatic black bears in southwestern China: high genetic diversity, broad host range, and zoonotic potential. *PLoS One* 12**,** e0171772.

Deng, L., Li, W., Zhong, Z., Gong, C., Liu, X., Huang, X., Xiao, L., Zhao, R., Wang, W., Feng, F., Zhang, Y., Hu, Y., Fu, H., He, M., Zhang, Y., Wu, K., and Peng, G. (2016b). Molecular characterization and multilocus genotypes of *Enterocytozoon bieneusi* among horses in southwestern China. *Parasit Vectors* 9**,** 561.

Feng, Y., Li, N., Dearen, T., Lobo, M.L., Matos, O., Cama, V., and Xiao, L. (2011). Development of a multilocus sequence typing tool for high-resolution genotyping of *Enterocytozoon bieneusi*. *Appl Environ Microbiol* 77**,** 4822-4828.

Karim, M.R., Wang, R., He, X., Zhang, L., Li, J., Rume, F.I., Dong, H., Qi, M., Jian, F., Zhang, S., Sun, M., Yang, G., Zou, F., Ning, C., and Xiao, L. (2014). Multilocus sequence typing of *Enterocytozoon bieneusi* in nonhuman primates in China. *Vet Parasitol* 200**,** 13-23.

Li, W., Cama, V., Akinbo, F.O., Ganguly, S., Kiulia, N.M., Zhang, X., and Xiao, L. (2013). Multilocus sequence typing of *Enterocytozoon bieneusi*: lack of geographic segregation and existence of genetically isolated sub-populations. *Infect Genet Evol* 14**,** 111-119.

Li, W., Cama, V., Feng, Y., Gilman, R.H., Bern, C., Zhang, X., and Xiao, L. (2012). Population genetic analysis of *Enterocytozoon bieneusi* in humans. *Int J Parasitol* 42**,** 287-293.

Li, W., Deng, L., Yu, X., Zhong, Z., Wang, Q., Liu, X., Niu, L., Xie, N., Deng, J., Lei, S., Wang, L., Gong, C., Zhou, Z., Hu, Y., Fu, H., Xu, H., Geng, Y., and Peng, G. (2016a). Multilocus genotypes and broad host-range of *Enterocytozoon bieneusi* in captive wildlife at zoological gardens in China. *Parasit Vectors* 9**,** 395.

Li, W., Wan, Q., Yu, Q., Yang, Y., Tao, W., Jiang, Y., and Xiao, L. (2016b). Genetic variation of mini- and microsatellites and a clonal structure in *Enterocytozoon bieneusi* population in foxes and raccoon dogs and population differentiation of the parasite between fur animals and humans. *Parasitol Res* 115**,** 2899-2904.

Wan, Q., Xiao, L., Zhang, X., Li, Y., Lu, Y., Song, M., and Li, W. (2016). Clonal evolution of *Enterocytozoon bieneusi* populations in swine and genetic differentiation in subpopulations between isolates from swine and humans. *PLoS Negl Trop Dis* 10**,** e0004966.

Zhao, G.H., Du, S.Z., Wang, H.B., Hu, X.F., Deng, M.J., Yu, S.K., Zhang, L.X., and Zhu, X.Q. (2015). First report of zoonotic *Cryptosporidium* spp., *Giardia intestinalis* and *Enterocytozoon bieneusi* in golden takins (*Budorcas taxicolor bedfordi*). *Infect Genet Evol* 34**,** 394-401.

Zhong, Z., Li, W., Deng, L., Song, Y., Wu, K., Tian, Y., Huang, X., Hu, Y., Fu, H., Geng, Y., Ren, Z., and Peng, G. (2017a). Multilocus genotyping of *Enterocytozoon bieneusi* derived from nonhuman primates in southwest China. *PLoS One* 12**,** e0176926.

Zhong, Z., Tian, Y., Song, Y., Deng, L., Li, J., Ren, Z., Ma, X., Gu, X., He, C., Geng, Y., and Peng, G. (2017b). Molecular characterization and multi-locus genotypes of *Enterocytozoon bieneusi* from captive red kangaroos (*Macropus rfus*) in Jiangsu province, China. *PLoS One* 12**,** e0183249.
